# A review of 3D printed medical implant design

**DOI:** 10.1186/s41205-025-00300-y

**Published:** 2026-01-22

**Authors:** Jatinder Madan, Paul Witherell, David W. Rosen

**Affiliations:** 1 Chandigarh College of Engineering and Technology (Degree Wing), Chandigarh, India; 2 National Institute of Standards and Technology, Gaithersburg, MD USA; 3 Institute of High Performance Computing, Agency for Science, Technology and Research (A*STAR), 1 Fusionopolis Way, #16-16 Connexis, Singapore, 138632 Republic of Singapore

**Keywords:** Customized, Implants, 3D printing (additive manufacturing), Design process, Variabilities

## Abstract

This paper reviews the design of customized 3D printed (also referred to as additively manufactured) implants. A focus is placed on the information flow of design as it is processed, starting from a patient’s scan and culminating with the 3D printing compatible customized implant’s design. We discuss the challenges related to the introduction of 3D printing technologies into the design of the implant, the variabilities encountered, and opportunities for standardization. The paper identifies research and standardization gaps in four stages of a 3D printed customized implant’s design process, namely, medical imaging, constructing CAD (3D) model of VOI, design, and 3D printing compatible file formatting. We hope the paper will help drive research to overcome future challenges encountered in the design process of 3D printed customized medical implants.

## Introduction

Most medical devices [1, 2] are prefabricated; this is less expensive than customized additive strategies and prefabricated devices are more readily available. The advantage of 3D Printing (additive manufacturing) is the ability to create a custom, patient-specific device on demand.

Customized implants provide an alternative solution to prefabricated devices, with the ability to meet many more of the anatomical and related requirements of a patient. In the present context ‘customized implants’ are those which are designed to match the patient’s unique anatomy and custom fit, which cannot be managed with the off-shelf components. Patient specific medical devices have been 3D printed for over 20 years and there are numerous benefits. For example, customized femoral implants result in 40% less bone removal at the bone-implant interface [1]. Customized chin implants demonstrate better anatomical and aesthetic outcomes than their prefabricated counterparts [2], whereas those for acetabular reconstruction showed excellent functional improvement and implant survivorship [3]. Furthermore, customized implants allow porosity and lattice structure which are helpful in enhancing biocompatibility [4, 5].

Patient specific 3D printed implants have been clinically applied for a variety of applications, such as craniofacial [6], mandibular [7], maxillofacial [8], orthopaedics [1, 9–15], cardiology [16] and, head and neck surgery [17, 18]. Despite comparatively higher costs and complexities when compared to off the shelf devices, the scope of patients for whom 3D printed devices are used continues to expand, albeit methodically. The quality of 3D printed customized implants is particularly important [19], with many researchers focusing on aspects related to material and manufacturing processes. Quality assurance challenges that account for digital and printing errors of 3D printed custom implants have been carefully studied [20]. However, less studied challenges are those associated with the design process of the 3D printed customized medical implants, a paramount activity that influences quality, cost, manufacturing and development times. The design process, in the present context, refers to the activities and information flow of design data as it is processed through various stages of a customized 3D printed medical implant’s design.

**Fig. 1:**
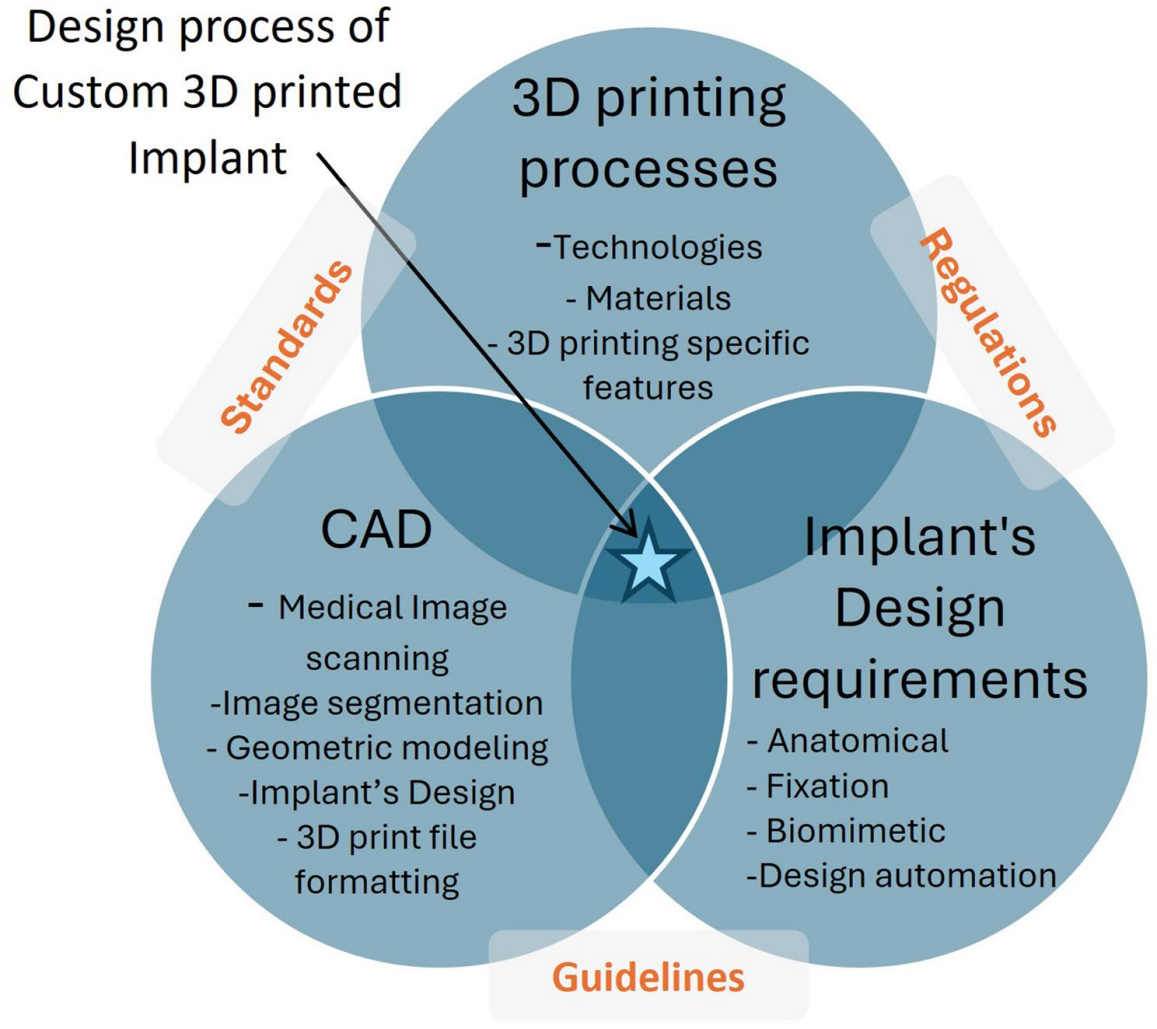
Key enabling technologies and concepts for 3D printed customized medical implants

3D Printed implants encounter variability during the design process, and there are consequences if these variabilities are not considered. This variability and the subsequent challenges are introduced from patient-specific conditions, the roles of physicians such as the radiologist, and engineers. Figure 1 illustrates the interaction between relevant technologies and concepts; from these arise two key research questions:

### RQ1

How is the patient specific *data processed*, what are the sources of variability, and who contributes to the research focused on this variability?

### RQ2

What *regulations*,* standards and guidelines* are important to custom implant design, and how are these being addressed?

The organization of this paper is as follows: Section “Methodology” presents the methodology adopted for conducting the literature search. Section “Reviewing the medical implant’s data flow” reviews medical implant data flow, from medical imaging to 3D print compatible file formatting. Section “Identification of research issues” discusses selected research issues. Section “Discussion on regulations, standards, and guidelines” provides a discussion on regulations, standards, and guidelines relevant to this study. Lastly, Section “Concluding remarks” presents concluding remarks.

## Methodology

Custom medical device designs are highly influenced by medical conditions and how they present in a specific patient. Traceability of the diagnosis and medical imaging information flows enables transparency and reduces discrepancies during the design and production process, a process that includes: the digital manipulation of a Volume of Interest (VOI), designing and preparing the implant’s CAD model of Final Anatomic Representation, the proceduralist’s (e.g. the surgeon’s) feedback on the model, file conversion to 3D printing compatible file format, 3D printing and post processing, and lastly inspection, deployment and post-surgery feedback [21, 22].

**Fig. 2:**
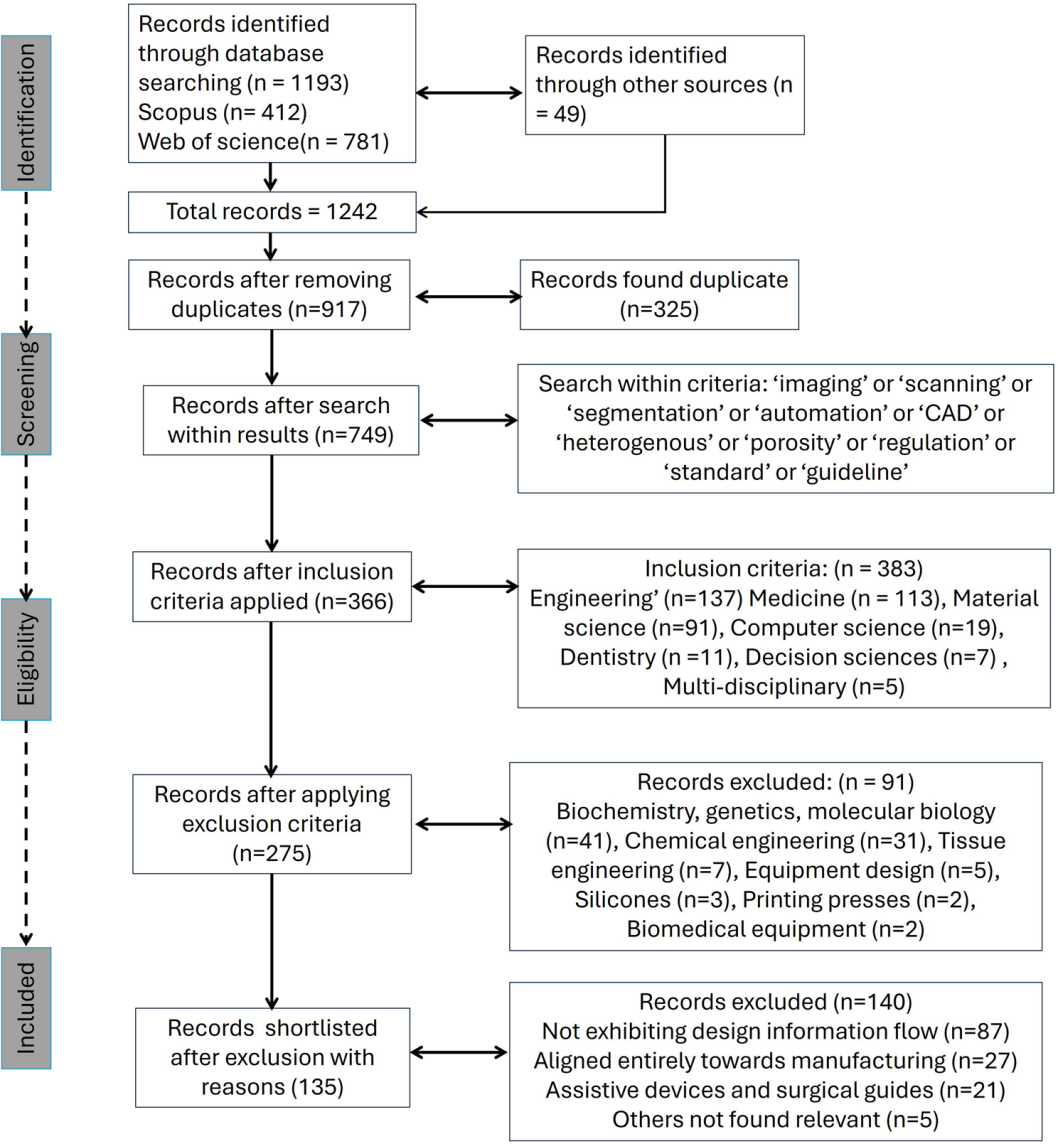
Literature search flow diagram

A systematic literature search (2001–2024) was made using Scopus and Web of Science abstracting databases by following these steps (Fig. 2):

- Keywords of ‘custom’ AND ‘medical’ AND ‘implant’ AND ‘additive’, AND ‘design’, resulted in 1242 documents. Synonyms of the above-mentioned keywords, such as ‘custom’, ‘patient-specific’, ‘patient matched’, ‘3D printing’ and ‘rapid prototyping’ were also used.
- Search within results was used to identify papers that discuss ‘imaging’, or ‘scanning’, or ‘segmentation’, or ‘automation’, or, ‘CAD’, or ‘heterogenous’, or ‘porosity’, or, ‘regulation’, or ‘standard’, or ‘guideline’.

The following inclusions were applied: ‘engineering’, ‘medicine’, ‘computer science’, ‘dentistry’, ‘decision sciences’, ‘multi-disciplinary’; the following exclusions were applied: ‘tissue engineering’, ‘equipment design’, ‘silicones’, printing presses’, ‘biomedical equipment’, ‘biochemistry’, ‘genetics’, ‘molecular biology’, and ‘chemical engineering’. The results were screened to a list of 135 relevant papers.

In this study, our focus is on the flow of information that originates with medical imaging and concludes with the fabrication of the part. Using the results of the literature survey to drive organization, the review is divided into three main parts:

(I) *Diagnosis*, which discusses acquisition of medical imaging data and manifestation of a representative CAD model. This includes the conversion of the scanned medical imaging data (2D) to a CAD (3D) model of the VOI.
(II) *Design*, which addresses the design and feature modification necessary to arrive at a suitable implant. This covers anatomy matching and design features that can be provided to the implant, focusing on the 3D printing compatible features.
(III) *Regulations*,* standards*,* and guidelines*, which are essential for making a 3D printed custom implant design acceptable.

## Reviewing the medical implant’s data flow

**Fig. 3:**
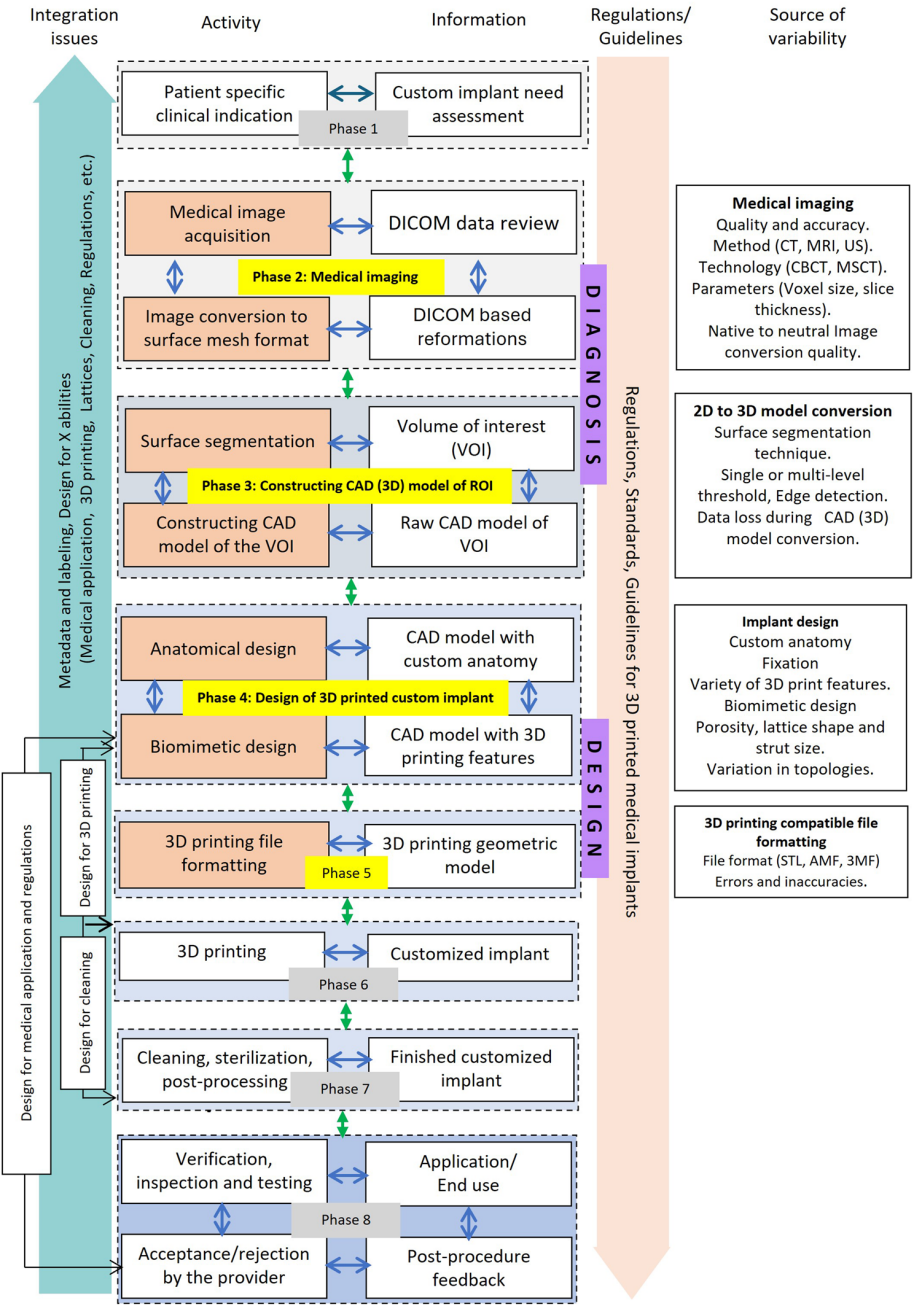
Information flow diagram for design process of custom 3D Printed medical implants

Figure 3 highlights information flows and representations from diagnosis to design to manufacturing to clinical use in different phases. Understanding the context and details at each phase, including data and metadata, and the transfer of design information between these phases, is critical for integrating a traceable information flow from design to manufacturing of 3D printed customized implants. The above-mentioned information flow is broadly divided into eight phases.

- *Phase 1* – Recognition of patient specific physical condition.
- *Phase 2* – Medical imaging.
- *Phase 3* – Constructing CAD (3D) model of VOI.
- *Phase 4* – Design of 3D printed custom implant.
- *Phase 5* – 3D printing compatible file formatting.
- *Phase 6* – 3D printing (additive manufacturing).
- *Phase 7* – Post-processing and cleaning.
- *Phase 8* – Verification, application and post-surgery follow-up, feedback.

With a focus on the design process, our review focuses on the role of the engineer from design to manufacture, thus sub-sections “Medical imaging” to “3D printing compatible file formatting” discuss the research issues associated with Phases 2 through 5 (highlighted in yellow in Fig. 3). The regulations, standards, and guidelines that govern the information flow of a custom 3D printed implant are considered important and are shown in Fig. 3 and discussed later in section “Discussion on regulations, standards, and guidelines”.

### Medical imaging

Two major steps of medical imaging are discussed in this section, namely (i) medical image scanning of an identified part of the patient’s body to collect data, and (ii) conversion of scanned imaging data to the neutral image format.

*Medical* imaging should be volumetric; it is used for diagnosis or to segment a volume of interest that will be used to create the patient-specific 3D printed implant. The large majority of patients have conventional volumetric CT images, although cone beam CT is still used – particularly for orthognathic procedures [23]. Understanding the capabilities of these systems is essential for achieving insight into the levels of accuracy and precision that are expected to be maintained throughout the information flow.

*Conversion of Digital Imaging and Communications in Medicine (DICOM) data to a surface mesh format for digital manipulation.* The DICOM file format was adopted in 1993 [24] and is the standard input from CT and MRI scanners. All modern conventional CT scans are volumetric, and most voxels are isotropic.

### Constructing CAD (3D) model of the VOI

The final anatomic representation is the digital surface mesh representation of a relevant VOI from the patient anatomy. It is a 3D model prepared using segmentation followed by constructing VOI’s CAD model.

#### Surface segmentation

Surface segmentation divides a 3D VOI to inform the design of the device. This is performed using automated segmentation methods as well as direct planimetry. The prominent automatic methods are global thresholding, multilevel thresholding, edge detection and region growing [25, 26]. Automated methods are ubiquitous, examples from MRI include the brain [27] and cartilage [28, 29]. The FDA has approved several software [30], most of which are equipped with multiple techniques for image segmentation.

Marked differences in accuracy of the segmentation methods have been found in literature. Reported accuracies range from 0.04 mm to 1.9 mm and the most commonly cited report on accuracy for global thresholding is 0.6 mm [25]. However, reported accuracies are subject to user adaptations. Huotilainen et al. [31] found major differences in the accuracy of STL files created for a patient undergoing tumor resection generated by three different institutions, with three different software preferences. It is also important to consider data accuracy within context. Essentially all custom 3D printed implants are derived from DICOM (CT or MRI) data. The accuracy of the DICOM data is typically on the order of 0.5 mm (or in some cases worse). Perhaps more important is the intended use of the device. For a cutting guide used for patient specific tumor resection, a typical surgical margin (the distance in any direction of healthy tissue surrounding the tumor that is measured ex vivo) is 10–20 millimeters. Thus, differences in accuracy on the order of a few millimeters likely have no clinical consequence.

#### Constructing CAD model of the VOI

The data obtained after surface segmentation needs to be further processed for constructing the CAD (3D) model of the VOI. The CAD model of the VOI is generally converted to an STL file, which has limited capabilities to truly represent heterogenous information of different anatomical regions. Furthermore, the level of accuracy of CAD model of the VOI obtained after segmentation may vary when representing different anatomical regions. For instance, the multi-threshold method provided an error of 0.14 mm in the proximal region of a long bone but an error of 0.30 mm in the distal articular region [26]. The extent of inaccuracy can be estimated to be about three times the voxel size. Typically a voxel size in image scanning of 0.35 mm may cause an error of about ± 1 mm [31].

### Design of custom 3D printed implants

Traditional design of a medical implant includes mechanical, biological, material, and thermal considerations. Further, the implant is also expected to exhibit chemical stability during its sterilization, and in-vitro and in-vivo conditions. A successful design carefully balances these considerations to meet patient-specific anatomy and custom fit requirements. The design of a custom 3D printed implant allows for additional considerations, namely customized anatomy, and the inclusion of biomimetic features, such as lattices and graded porosity. This section explores how these 3D printing -specific considerations are being addressed.

#### Customized anatomical design

The fundamental advantage of 3D printing is that the anatomy is patient specific. However, truly realizing the advantages requires tight design processing from the volumetric medical images [32]. Furthermore, the disease (e.g. trauma) impacts the VOI, and the model created should be free from damage or defects. This poses several challenges and requires reconstruction techniques such as mirroring and free form modeling. These are routinely used for cranial and mandibular implants [6, 33, 34].

Several steps can be automated. For example, Hieu et al. [35] developed a system for the design of cranioplasty implants for skull reconstruction surgeries. Vignesh et al. [36] reported reconstruction of late post traumatic orbital wall defect by creating a CAD model of the defected zone, taking advantage of symmetrical features. Chaudhary et al. [37] developed an algorithm for automatic realignment of unhealthy anatomies in various musculoskeletal defects that require reconstruction surgeries. Burge et al. [38, 39] applied techniques of machine learning and CNN to automate customized pipelines for knee implants so that the implant design matches the patient’s anatomy. The major commercial software packages offer a suite of tools, and some software packages are customized for a specific clinical scenario. While these tools can successfully automate some of the design process, the printed part still requires substantial engineering work and consultation with the proceduralist. Surgeons and engineers should collaborate during different stages [40], facilitating digital workflows [41], and guiding the design of customized medical implants [42–44].

#### Biomimetic design

Biomimetic design refers to emulating the human properties required for form and function of the 3D printed parts. Lattices, graded porosity, and complex contours can be designed for 3D printing; these are generally not feasible with conventional manufacturing.

Lattices are a sub-set of one or more categories of cellular solids, which include naturally occurring structures, such as honeycomb, cancellous bone, and sponge [45]. Lattices provide the advantages of controlling relative elastic modulus and volume fractions. These are essential for implants; correct designs promote osteointegration, reducing stress shielding and reducing sensitivity by reducing thermal conduction.

3D printing processes help improve desirable properties by facilitating the anatomical correctness of a customized medical implant and managing porosity [46]. Graded porosity [47, 48] allows for varying density and stiffness to reduce stress shielding. Sutradhar et al. [49] applied topology optimization to design an implant that remains anatomically matched but with an improved strength to weight ratio. To facilitate osseointegration and improve performance [50], Shi et al. [51] augmented material selection with improved design features using designed porosity and topology optimization. Poh et al. [52] optimized the performance of a bone scaffold using appropriate porosity distribution. Vu et al. [53] studied the effects of surface area and topography on 3D printed scaffolds for bone grafting applications. These efforts are indicative of the increasing flexibilities offered in 3D printed custom implant design.

Design and CAD modeling of 3D printed implants with features like lattices and graded porosity often follows a tedious workflow of selecting appropriate lattice structure (BCC, FCC, etc.) and unit cell type (primitive, gyroid, diamond, etc.), lattice geometric parameters, filling space, and grading scheme. Designing an implant with such features can be very challenging, costly and time consuming. However, with the availability of implicit CAD modeling software [54–56] the design workflow can now be better managed in comparison to the use of parametric CAD software [57–59].

There are several reports of design automation that include features such as lattices and graded porosity. Thomas et al. [60] use a variety of unit cells to prepare graded lattice structures to identify and optimize a customized 3D printed implant’s performance. Naghavi et al. [61] use implicit modelling to design and simulate a customized 3D printed hip implant using TPMS unit cells. They demonstrated that, in comparison to a solid stem, a porous stem shows significantly improved performance in terms of stress shielding, bone resorption and bone loss reduction. Guariento [62] developed an algorithm that uses a Gyroid unit cell and the nTop system [54, 56] to rapidly generate CAD model of a pelvic prosthesis while maintaining desired biomechanical properties of the implant. El-Gizawy et al. [63] proposed an approach to help design a 3D printed customized implant for joint reconstruction applications. This approach was tailored to mimic bone anisotropic properties and microarchitectures. Specialized workflows developed by other commercial establishments use implicit modeling to design 3D printed customized implants with lattice structures [64]. Dayanç et al. [65] automated design of an implant with lattice structures using implicit modeling technique for some applications like humerus bone fracture.

**Fig. 4:**
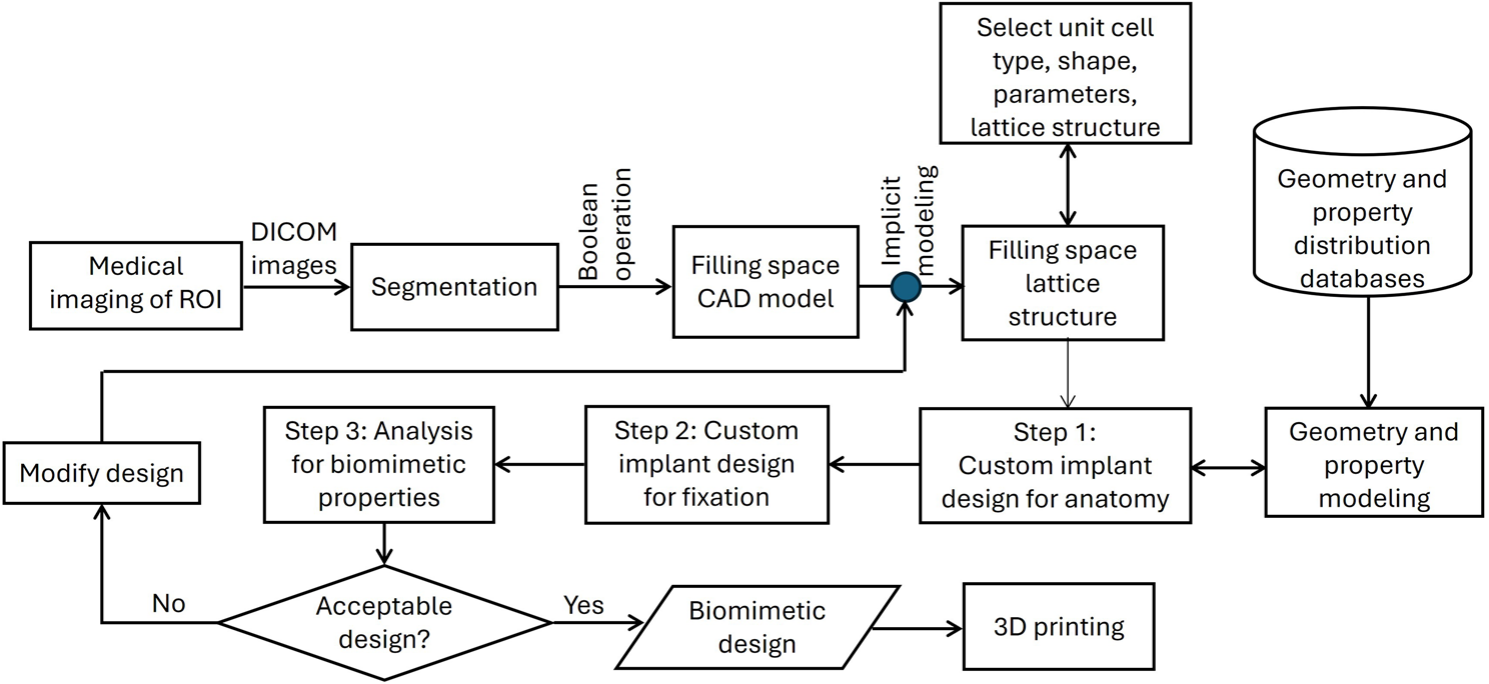
A typical workflow of 3D printed customized bone implant design with lattice structures using implicit modeling

Figure 4 presents a design workflow for bone with features of lattice structures developed using implicit modeling techniques. DICOM data that includes the VOI are converted into a segmented CAD model (boundary representation or B-rep). A Boolean operation is applied between CAD models (B-rep) of the healthy bone and the pathology to get a CAD model of the filling space for the implant. Implicit modeling is applied on the CAD model of the filling space to provide porous lattice structure by selecting appropriate unit cell type, shape, and other required parameters [66]. The filling space CAD model then has characteristics that resemble bone properties, such as stress shielding, elastic modulus, and relative density. The prepared implicit model meets the anatomical requirements of the bone but still requires additional features for integration. For example, in a pathologic fracture, to join the remaining two fractured parts of the bone. The design for this clinical scenario will include one or more plates to cover the pathology with porosity and holes to allow for the joining of the implant with screws. The implicit model of the implant is analyzed to measure its biomimetic properties for comparison with the design requirements. The design iterations are made till satisfactory design of the implant is prepared.

### 3D printing compatible file formatting

In preparation for fabrication, the design model of a 3D printed custom implant must be converted into a compatible file format, most often tessellated, and thereafter sliced into planar (horizontal) layers. Common file formats compatible [23] with 3D printing build requirements [67] include stereo-lithography (.STL), additive manufacturing format (.AMF) and 3D manufacturing (.3MF) [68].

The *STL file format*, developed in 1987 [69], is considered to be the industry standard and remains popular [70]. The STL file is a representation of boundary surfaces, each surface as a combination of several triangles, and is thus an inexact representation. Challenges often encountered with STL files include inaccuracies due to approximation of the surface with triangulation, massive file sizes for parts with complex features, and an inability to represent material and other properties of the target object [71]. Research efforts to represent heterogeneous materials, such as density distribution in a human bone, are indicative of the challenging requirements in finding suitable file formats [72–75].

The *AMF format* is an XML based open standard published as ISO/ASTM 52915:2016 for describing objects for AM (or 3D printing) processes [76]. Unlike STL, AMF has native support for color, materials, and lattices. AMF allows regions of the part to be defined geometrically either using a triangle mesh or through a voxel bitmap. The bitmaps allow for each region to be associated with a distinct material [77]. The format is beneficial as it supports multiple materials, functionally graded materials, and meta-materials as a functional combination of two previously defined materials. AMF has the capability to represent microstructures with relatively low file size [76].

The *3MF format*, the result of an industry consortium to address file format challenges, makes use of XML and other technologies to describe the content and appearance of 3D models largely driven by the advancements in 3D printing technology [78, 88]. Like AMF, the format supports efficient storage of lattices and multi-material, while also being considered human readable, simple, extensible, unambiguous and free [79]. Furthermore, extensions of the 3MF format can support additional material properties, slicing, and production.

## Identification of research issues

This section discusses emerging and ongoing research issues and opportunities at each of the stages previously described.

### Medical imaging

Imaging modalities have varying spatial resolution, contrast resolution, and signal to noise properties [65, 66, 80–84]. Most 3D printed parts begin with CT, and this can be either cone-beam or conventional. Within a modality (e.g. among CT), there are varying *scanning parameters*, such as slice thickness, field of view (FOV) and reconstruction algorithms [24, 31, 80, 83, 85–87]. These are detailed with recommendations for image acquisition suitable for 3D printing in [23]. When the images are compressed for communication, the workflow should minimize variabilities and loss of data [88, 89].

Because DICOM does not have clear operational definitions and guidelines for identifying landmarks [90], input from the radiologist and proceduralist (if different from the radiologist) is essential for identifying anatomical landmarks [91]. Surface segmentation converts intensity-based information within the VOI to construct a B-rep model; which results in loss of heterogeneous medical data [83]. On the other hand, 3D printing technologies have demonstrated the capability to produce heterogenous medical parts, such as variable densities in cortical and trabecular regions of a bone [92, 93]. Because there is a loss in heterogeneity in most workflows, a credible method that helps retain heterogeneous information will enhance custom implants in patients for whom that heterogeneity is important in the pathology and repair. Finally, there may be a need to convert images from proprietary formats to DICOM [90, 94], and this conversion does not have standardized validation procedures. Table 1 summarizes the identified research issues relating to medical imaging for 3D printed custom medical implants.

**Table 1:** Identified research issues relating to medical imaging

Sub-topic	Summary of the research Issue
Quality of imaging data	- Different modalities have unique properties such as spatial resolution.
	- Different scanning technologies (such as CBCT versus conventional CT) in theory can have differences. These have been largely mitigated with new hardware platforms.
	- Variations in scanning parameters.
	- Lack of guidelines for acquiring, communicating, and archiving images.
Anatomical landmarks	- Challenges in the identification of anatomical landmarks.
	- Automation tools to identify anatomical landmarks have variability.
Heterogenous medical data	- Lack of methods for capturing imaging heterogeneity when it is medically important.
Validation of DICOM data	- Lack of validation procedures for converting non-DICOM data to the DICOM format.

### Constructing CAD (3D) model of the VOI

Accuracy and consistency in the CAD model VOI is vital [88]. An example is an orbital floor implant that requires high precision [95]. The following research issues relate to variabilities in the DICOM to CAD model conversion.

#### Surface segmentation

In practice, surface segmentation is done using automated systems such as thresholding [96], and then cleaning up the model with direct planimetry. The latter requires high expertise and is time consuming and costly [25, 89, 97]. The segmented models typically have holes and burrs [98], creating additional cost and time commitments. There is enthusiasm for deep learning techniques to enhance the workflow for custom implants [25, 89]; specifically, these strategies are slated to improve accuracy and consistency as well as providing time-savings.

*Verification and validation* of both the segmented data and the resulting CAD model of the VOI is important for the design process. S*tandard guidelines and best practices* are important benchmarks for CAD validation. Mean surface deviation provides a global perspective of the implant’s accuracy but lacks in capturing the local inaccuracies. New procedures for verification of the segmented data must include local and anatomy specific deviations (such as by providing ‘heat map’ or using different colors) [25]. Biomimetic phantoms are needed to support the validation of segmentation [99]. Differences in scanning parameters [36, 100] may also cause inconsistency in the CAD model of the VOI.

#### Retaining heterogeneous information

Heterogeneity is a basic characteristic of implantable body parts, such as human bone. However, available techniques lack the capability to accurately represent biomimetic heterogeneity at the surface segmentation stage and later when the CAD model of the VOI is prepared. Therefore, future research efforts should focus on developing new schemas and representation methods [101], and tools that help convert and retain heterogeneous information [102].

Table 2 summarizes the research issues related to the construction of a CAD model from the VOI for a 3D Printed custom medical implant.

**Table 2:** Identified research issues relating to constructing CAD model of the VOI

Preparing CAD (3D) model of the VOI	
Sub-topic	Identified research issue
Image segmentation	- Manual segmentation methods are exceedingly strenuous, costly, and time consuming. - Inadequacies and inconsistencies are pervasive in automated segmentation methods
	- Lack of automated error correction methods for holes and burrs.
V&V of segmentation techniques	- Lack of standard test methods, including the use of biomimetic phantoms, to validate segmentation techniques.
	- Insufficient metrics for the global and local verification of segmented data.
V&V of VOI’s CAD model	- Lack of standard methods to validate conformance of a CAD model to the VOI
Retaining heterogenous information	- Lack of capabilities to capture biological heterogeneity and therefore emulate human tissues

### Design of custom implants

The conversion of a VOI’s CAD model to the design of a customized implant is a cumbersome process that involves significant efforts from referring providers, radiologists, and engineers. The following are identified as design research issues.

#### Anatomical design

Creating a perfected model is challenging for patients with significant pathology [103, 104], which makes the design process cumbersome, are used for anatomical design. Techniques such as the mirror image technique, twin plate spline, free form modeling, template-based technique, statistical shape models, and snakes’ anatomical design highlight ongoing research efforts. However, these techniques are based on largely automated unguided heuristics with unquantified limited scopes. Even though they are commonly used, the technology is nascent [33, 34].

#### Biomimetic implants

Lattices, graded porosity and topology optimization have been used to improve the custom implant’s biomimetic characteristics [65, 105, 106]. Despite useful research attempts discussed in the previous section (refer to sub-section on Biomimetic design), research challenges in the design process of biomimetic 3D printed custom implants with lattices and graded porosity remain. First, due to the limitations of the parametric CAD modeling, techniques like triply periodic minimal surface (TPMS) [107] and Voronoi tessellation are useful in providing porosity and functional grading in 3D printed custom implants [108–110] with relative ease. However, such features are not commonly employed by the medical community [65] primarily due to lack of expertise of implicit modeling and tedious design workflows. Second, the design of an implant requires several non-trivial tasks, such as selecting an appropriate lattice structure and deciding its parameters, internal structure and topology optimization. This requires developing characterization and empirical models to assess the expected performance of an implant’s design in an efficient manner.

#### Representing heterogeneity in CAD models

Meta-materials [9, 111, 112] and functionally graded materials [48, 113] are being developed to improve in-vivo performance by shifting the design approach from contour modeling to performance modeling. However, the CAD technologies that can define heterogenous and anisotropic materials are still in their nascent stages [92, 101, 102, 113–116], whereas, 3D printing of such biomaterials is advancing rapidly [92, 93, 117]. Although implicit modeling approaches provide partial solutions, their adoption is constrained by complex workflows and limited user expertise.

#### Terminology

Inconsistent terminologies and attributes are used to describe the design features of 3D printed medical implants. For instance, “porosity” is commonly used to describe the ratio of void space to the total implant volume in the medical community and is a desirable aspect [118, 119]. However, “porosity” for other engineering components is often used to describe weakness and is undesirable. Presently, the medical community uses standards for porous coatings with limited scope for 3D printed medical implants, particularly for lattice structure-based parts that are porous throughout their volume. New, consistent terminology specific to the need of implants is required to provide consistency in the description of their design features.

#### Sterilization and cleaning

3D printed process capabilities enable designers of custom implants to use features like lattices for attaining desirable characteristics such as porosity as well as apply topology [49] and structural optimization [120]. However, the subsequent intricate details often make the cleaning and sterilization preparation of 3D printed custom implants difficult. Therefore, systems that help design 3D printed implants which facilitate cleanliness, mandatory sterilization, and testing, are lacking [34] and in need of further research efforts.

#### Design automation

Research on design automation [19, 113, 116, 121, 122] has largely concentrated on form design. In contrast, the development of custom implants requires additional automated support for decisions on material selection, anatomical adaptation, fixation strategies, biomimetic features, and design-for-X (DfX) considerations. Although notable progress has been achieved in automating custom implant design [122–126], the incorporation of DfX aspects—such as cleaning and sterilization—remains insufficient.

#### Verification and validation

Once the design of an implant is completed, its V&V to the VOI and other requirements are a necessity. Although regulations make it mandatory to provide key parameters (e.g., GD&T, porosity, compressive stiffness) for design V&V [127], the absence of well accepted metrics leaves V&V ill-scoped and open to subjectivity and personal preferences. The design of custom 3D printed implants needs more reliable metrics to verify and ascertain conformance to anatomical and biomimetic design requirements.

#### Guidelines, training and certification

Tedious workflows require much effort from the physicians and engineers especially when biomimetic features like lattices and graded porosity are to be provided. Furthermore, additional design requirements for customized 3D printed implants catering to different medical specialties pose additional challenges. Despite research attempts to automate the design workflow, the role of physicians and engineers cannot be ignored. Therefore, guidelines that help to meet the design requirements are necessary [65, 103, 104]. Further, training and certification of the personnel involved is highly desirable so that they can follow established procedures, guidelines and also meet regulatory requirements [128, 129].

Table 3 summarizes the research issues relating to the design of 3D printed custom implants.

**Table 3:** Identified research issues relating to the design of custom 3D printed implants

Design of customized medical implant	
Sub-topic	Summary of Issue
Anatomical design	- Lack of automation for converting VOI’s CAD model to an acceptable anatomical design. - Immature methods for assisted and automatic repair of a damaged or unacceptable VOI model.
	- Lack of guidelines for preparing anatomical design for acceptance.
Biomimetic design	- Lack of expertise in implicit modeling and tedious design workflows. - Insufficient CAD modeling capabilities for complex biological representations, such as graded porosity and lattices. - Characterization and empirical models for assessing expected performance of the implants with lattices, graded porosity considering its structural and topology optimization.
Representing heterogeneity in CAD models	- Insufficient technologies for representing the inherent heterogeneity of biological components in their CAD models. - Lacking capability of CAD modeling of heterogenous materials.
Terminology	- Absence of common terminology references and standards for representing design features of 3D printed implants.
Sterilization and cleaning	- Lack of guidelines and system support for designing implants for sterilization and cleaning.
Design automation	- Lack of system support for design considering range of design parameters and requirements, such as materials, anatomy, fixation, biomimetic features, and design for x-abilities.
Verification and validation	- Need for additional metrics to facilitate V&V, acceptance and conformity of the final 3D printed custom implant to the patient.
Guidelines, training and certification	- Guidelines for navigating implant design workflow considering biomimetic features alongside defining roles of physicians and engineers. - Guidelines for implants meant for medical specialties. - Training and certification of the personnel.

### 3D printing compatible file formatting

The STL file format is the most common way of transferring the implant design data for 3D printing. However, STL file formats face several problems such as a lack of multi-material support [68, 70, 77]. Furthermore, representing implants that have several very small features, like lattices, results in a very large STL file. Although significant progress in this context has been made by multi-organization efforts like AMF [68] and 3MF [78, 79], format adaptability in the industry is still miniscule.

The AMF and 3MF file formats can support desirable features like meso-structures and lattices, which are extensively used in the custom implants to make them biomimetic. However, their effectiveness depends on the quality of design data available in the CAD model file, where representations are still lacking in the ability to represent anisotropic, heterogeneous and multifunctional meso-structures [110, 116, 130].

Research attempts are being made to make biomimetic implants which can replicate the internal structure of natural body parts. These internal structures possess distinct properties such as porosity, functional grading, meso-structures, and hierarchical forms that are often lost during medical imaging [108]. Implants with such internal details can only be made if additional information is maintained through all stages of the design process, including the last stage of conversion to the 3D printing compatible file format. Furthermore, 3D printing process requires slicing of the 3D printing compatible file format. However, available sliced models don’t have capability to directly handle such heterogenous information [131]. Therefore, consistent research efforts that address the design data integrity requirements highlighted throughout the information flow pipeline of 3D printed custom medical implants are highly desirable.

Table 4 summarizes the research issues related to the conversion of CAD data to 3D printed compatible file formats.

**Table 4:** Identified research issues relevant to converting the implant’s CAD model to 3D printed compatible file format

3D printingd compatible file formatting	
Sub-topic	Summary of issue
Adoption of new 3D printing compatible file formats	- Need for guidance and best practices to support broader adoption of enhanced file formats such as 3MF and AMF in lieu of STL.
Design data integrity requirements	- Maintaining design data integrity during all stages of the design process, including the last stage of conversion to 3D printing compatible file. - Handling heterogeneity information during slicing of the 3D printing compatible file. - Need for reference workflow to showcase retention of heterogenous medical data as it is translated across design information flow pipeline.
Quality of CAD data received	- Availability of anisotropic, heterogeneous and multifunctional meso-structures information.

## Discussion on regulations, standards, and guidelines

Regulations, standards, and guidelines play a crucial role in ensuring consistency and minimizing variabilities in design and manufacturing. The perceived inadequacy of suitable regulations and guidance is considered by some to be a hindrance in growth of the 3D printed customized implant industry [118, 132]. A larger cohort agree that more widespread adoption would follow from the development of more suitable standards [133] to ensure the greatest benefit from 3D printed implants [134].

### Addressing barriers to trade

Some standards and regulations, due to their geographical jurisdictions, act as barriers to trade. Recognizing these obstacles, the WHO has constituted the Global Harmonization Task Force (GHTF). The GHTF, represented by Australia, Canada, Japan, European Union, and United States of America, works for convergence in standards and regulatory practices for medical devices. In the case of 3D printed custom implants, not all jurisdictions have yet adjusted their regulatory framework to accommodate their adoption. The widespread maturing of 3D printing within the biomedical industry will ultimately be dependent on the synthesis of regulations and technology [135] to promote accessibility.

The International Medical Device Regulators Forum (IMDRF), an offspring of the GHTF with several other countries joining in, has begun to introduce regulations for 3D printed custom implants [127, 136, 137]. To support transparency, these regulations mandate that clinicians and manufacturers record design details like imaging parameters, structural parameters (such as geometry, porosity, lattice strut size) and performance parameters (such as density, and compressive stiffness) [130, 138]. Several of the regulatory pathways applicable to custom 3D printed implants are also applicable for special circumstances like those involving pediatric and young patients [139] and must be subject to rigorous testing and validation [140]. The implants for such special circumstances should also fulfil the requirements of patient’s growth and development, biocompatibility and safety [141].

### Terminology and metrics

Gaps remain in areas where efforts could have significant impact, including commonly accepted terminology and metrics for imaging parameters, porosity, lattice strut size, wall thickness as well as performance parameters like density, and compressive stiffness.

### Design guidelines

Guidelines help ascertain adherence to well-established procedures, workflows, and quality parameters. While some 3D printing guidelines are available, they lack the specifics for stakeholders to navigate the design procedures for 3D printed custom implants. Standards development organizations including ASTM International and ISO have significant efforts underway, particularly in ASTM committee F42 and ISO TC 261, both focused on 3D printing technologies. ASTM and ISO have a partnership agreement that fosters close collaboration on standards development. For the issues identified in section “Design of custom implants”, initial discussions have occurred in the ISO/ASTM joint group JG54, on design guidelines, with the ASTM F42.07 medical device group to identify opportunities for design guides for medical implants. To promote maximum use of additive manufacturing (or 3D printing), the US FDA has issued guidelines for technical consideration for medical devices made with 3D printing technology [142]. These guidelines, however, remain generic and research efforts that help establish design quality [91, 143, 144] should contribute to the development of new guidelines (or procedures) to ensure consistent workflows.

### Standardization efforts

In a recent development, the American National Standards Institute (ANSI) [99] provided the next roadmap for developing 3D printing standards, identifying medical applications as one of the major focus areas. The roadmap identifies important gaps related to consistency and accuracy of medical imaging data as well as its processing focusing on three major aspects discussed in this paper, namely imaging data, segmentation of imaging data and design of lattice structures. In addition to ASTM F42 and ISO TC 261, ISO TC 210 focuses on quality management and corresponding general aspects for medical devices. In the dentistry area, ISO TC 106 has a standard under development on accuracy of polymer dental products fabricated with vat photopolymerization processes [145]. In ASTM F42, the F42.07 subcommittee on applications has nine focus areas, including medical devices.

### Cleaning and sterilization

Cleaning and sterilization are critical for all implants, but they are especially important for 3D printed-customized implants, which must be free from loose particles and foreign matter while meeting established sterilization criteria [146]. This necessity arises primarily from their unique design features, such as porosity and lattice structures. Therefore, guidelines are needed to address design considerations during the early stages of 3D printed custom implant development for effective cleaning [99]. The ASTM International committee F04 on Medical and Surgical Materials and Devices has developed standard guide F3335-20 for assessment of residual powder after PBF processing.

### Clinical and biological evaluation

The ISO TC 194 committee, which oversees the clinical and biological evaluation of medical devices has developed a series of standards (ISO 10993-1 through 23) on biological evaluation of medical devices [147] focused on various considerations and methods, test procedures, toxicity and various hazards, sterilization, etc.

### Collaborative standardization efforts

Since the 3D printing of medical implants is a highly interdisciplinary topic, various collaborations among technical committees in the standards community have been formed. A unique Partner Standards Development Organization (PSDO) Cooperative Agreement was established in 2012 between ISO TC 261 and ASTM F42 to promote the co-development of AM standards. In ASTM International, committee F04 has two standards directly related to 3D printing, ASTM F3335-20, already mentioned, on the removal of 3D printing residues in medical devices [148] and another standard guide on bioinks used in bioprinting [149]. They have also developed many material specifications for medical devices and implants, but these generally are not limited to 3D printing processing. Rather, they specify properties and characteristics that a material type should have regardless of manufacturing process. Typically, biocompatibility, cleaning procedures, and sterilization characteristics are specified. ASTM Committee F42 have established collaborations with ASTM Committee F04 on Medical and Surgical Materials and Devices. Additionally, several joint groups have been formed between ASTM F42 and ISO TC261 in the medical area. For example, joint group JG70 was established to focus on medical imaging data and its relationship to 3D printing processes [150]. Issues identified in Sect. 4.1 and 4.2 are within scope, including imaging resolution, neutral image conversion, image quality, segmentation, VOI identification, and CAD model development.

*Standards for 3D printing (AM*) *file formatting*: Joint group JG64 focuses on the AMF file format; a significant portion of their efforts address the medical image and model representation issues identified in Section 4.4, including the capabilities of AMF’s voxel representations and other solid modelling capabilities.

### Standards for design data

At present, a design guide on post-processing of metal parts was developed in JG54 [151], part of which addresses issues of relevance to metal implants [130, 138]. Additional activities between ISO and ASTM focus on the content and format of data packages for communicating design information to manufacturing organizations, in JG73, and an overview of 3D printing data processing, which are both relevant to 3D printed custom medical implants. Additionally, emerging issues related to the legal role of CAD model files and data protections have the potential to create liability related issues [118], which need proper addressal.

*Training and certification*: The successful design of a custom implant requires the multidisciplinary skillsets of design, manufacturing, standards and regulations, as well as greater attention to the training and certification of personnel [128, 129, 152]. Given the crucial roles of the equipment operators, and the manpower involved in the design of custom medical implants, new specialized trainings are essential. Basic questions that should be considered in this context are: Who should be the designer? How should they be trained? How should they be certified as competent? Further consideration of design and quality standards such as ISO13485 [153] may provide an answer, a solution increasingly being achieved by hospitals in Europe.

Together, these different organizations are beginning to address many of the issues outlined in this paper, but many continue to remain unaddressed.

## Concluding remarks

3D printed custom implants have enhanced – and for some patients revolutionized, care pathways. Specifically, 3D printing offers a promising approach for creating custom medical implants with intricate details and desirable features that are often unachievable with traditional manufacturing methods. The manufacturing of an implant requires a 3D printing compatible file for the implant which is obtained only after a tedious design process comprising several phases. In this paper we discussed various challenges that arise throughout their design process, primarily due to the need for design data processing at multiple stages and the subjective decisions made at different levels. The literature reviewed in this paper covers four key stages in the design of 3D-printed customized implants: medical imaging, construction of the CAD model of the VOI, design of the custom implant, and formatting of files compatible with 3D printing. The research challenges encountered at each of these stages, which must be addressed to reduce variability and enhance consistency in the design process, are also discussed. Additional challenges stem from the advanced capabilities of the 3D printing technologies, which can now handle materials like functionally graded, heterogenous, lattices and graded porosity. However, the technologies employed for design processing have not advanced sufficiently to provide required data for 3D printing processes. The discussion also included regulations, standards and guidelines related to the design of 3D printed customized medical implants.

**Fig. 5:**
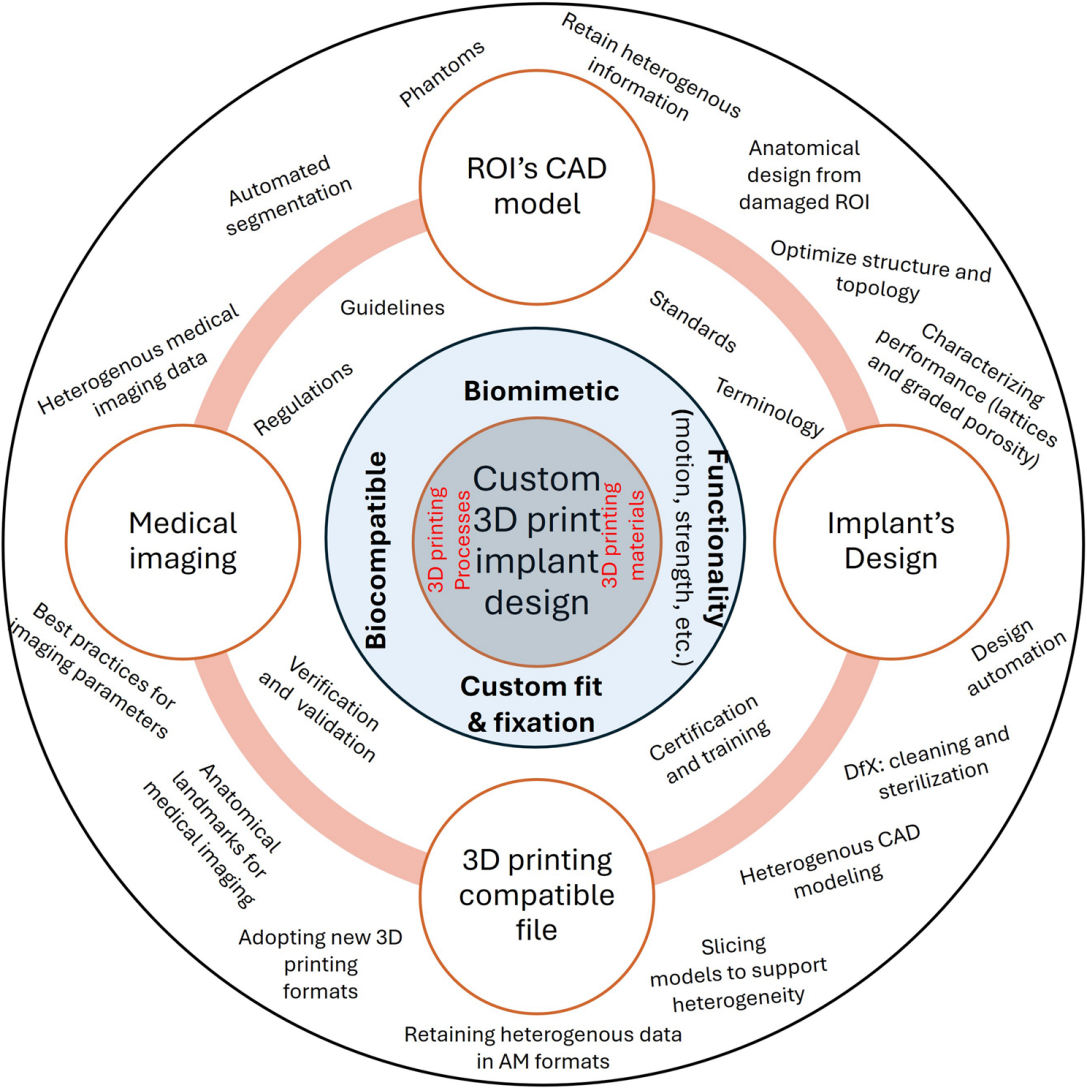
Conceptual illustration of design process of customized 3D printed medical implants

Figure 5 illustrates the key research challenges associated with designing customized 3D printed medical implants. At the core of the figure is the implant design, which is constrained by the 3D printing processes and materials, highlighted in red. Surrounding the core are four critical design aspects: biocompatibility, biomimetic, functionality, and custom fit & fixation. The outermost layer represents the four important phases of the design process focus of this paper, with research issues specific to each phase displayed alongside. Additionally, the middle layer highlights broader research issues that are relevant across all phases of the design process.

### Disclaimer

Mention of commercial products or services in this paper does not imply approval or endorsement by NIST, nor does it imply that such products or services are necessarily the best available for the purpose.

## Data Availability

No datasets were generated or analysed during the current study.

## Acronyms

AM: Additive Manufacturing
CAD: Computer-aided design
CAM: Computer-aided manufacturing
FDA: United States Food and Drug Administration
VOI: Volume of interest
STL: Stereolithography
3MF: 3D manufacturing format
CNN: Convolutional neural networks
TPMS: Triply periodic minimal surfaces
BCC: Body centred cubic
FCC: Face centred cubic
GD&T: Geometric dimensioning and tolerancing
CNC: Computer numerical control
3D: Three dimensional
CT: Computed tomography
CBCT: Cone-beam computed tomography
MSCT: Multi-slice spiral computed tomography
AMF: Additive manufacturing format
MRI: Magnetic resonance imaging
DICOM: Digital Imaging and Communications in Medicine
XML: Extensible Markup Language
NIFTI: Neuroimaging Informatics Technology Initiative
V&V: Verification and validation
DfX: Design for x-abilities
